# Editorial: Integrated diagnostics and biomarker discovery in endocrinology and biomedical sciences, volume II

**DOI:** 10.3389/fendo.2025.1739424

**Published:** 2025-11-21

**Authors:** Kinam Park, Mikyung Jin, Yong-Min Kwon, Yena Park, Soon-Yong Kwon, Sajung Yun, Youngmi Ji, Sijung Yun

**Affiliations:** 1 Predictive AI, Inc., Seongnam, Republic of Korea; 2 Department of Radiology, Youido St. Mary’s Hospital, Catholic University of Korea, Seoul, Republic of Korea; 3 Seoul Bethesda Hospital, Seoul, Republic of Korea; 4 Johns Hopkins University, Baltimore, MD, United States; 5 US-Asia Technology Management Center, Stanford University, Stanford, CA, United States; 6 National Institute of Dental and Craniofacial Research, National Institutes of Health, Bethesda, MD, United States; 7 Predictiv Care, Inc., Mountain View, CA, United States

**Keywords:** multi-omics, systems endocrinology, precision medicine, biomarker discovery, integrative analysis, endocrine disorders

The integration of multi-omics biological data, such as genomics, transcriptomics, proteomics, etc., is reshaping how we conceptualize and pursue biomarker discovery in endocrinology. Moving beyond reductionist paradigms, contemporary research now unites molecular, cellular, physiological, and population-level information to illuminate the complex regulatory architecture underlying endocrine health and disease. Integrated Diagnostics and Biomarker Discovery in Endocrinology and Biomedical Sciences: Volume II brings together nine original contributions that exemplify this transition toward a systems-oriented and data-driven discipline.

Spanning the spectrum from ionic ratios and proteomic signaling to transcriptomic networks, genomic variation, and ecological microbiome interactions, these studies demonstrate how diagnostic precision emerges through the convergence of molecular and systemic perspectives. Collectively, they trace a coherent trajectory - from basal biochemistry and molecular communication to clinical integration and population-scale modeling - illustrating how multi-scale data synthesis from ionic ratios to networks can refine both mechanistic understanding and translational application.

Taken together, this Research Topic reflects the growing maturity of integrative endocrinology, a field where multi-omics analytics, causal inference, and real-world data harmonization converge to enable predictive and personalized approaches to endocrine disorders. By highlighting these multi-scale insights, Volume II underscores the central message of modern biomarker science: meaningful diagnostic innovation arises not from any single data layer, but from their integration into a unified systems framework that connects molecules to medicine.

Lou et al. systematically validated the Glucose–Potassium Ratio (GPR) - a long-recognized broad clinical predictive marker (1) - as a prognostic biomarker for both short- and long-term all-cause mortality. They showed a strong association with mortality in both hospital and ICU settings. Mortality risk escalated sharply when GPR exceeded this threshold. Sensitivity analyses confirmed the robustness of these findings, positioning GPR as a valuable, non-invasive indicator for early identification and risk stratification of high-risk sepsis patients. This study opens the Research Topic by illustrating that integrated diagnostics can arise not only from macromolecular data but from fundamental ionic interactions reflecting systemic metabolic homeostasis.

Ji et al. utilized Tandem Mass Tag (TMT)-based quantitative proteomics on serum-derived exosomes to compare protein profiles among juvenile gout (J-Gout), juvenile hyperuricemia (J-HUA), and oligoarticular juvenile idiopathic arthritis (oJIA) patients. Subsequent ELISA validation confirmed that two proteins’ concentrations were significantly high in J-Gout. Furthermore, their marker levels showed a positive correlation with clinical inflammatory indicators, C-reactive protein (CRP), and erythrocyte sedimentation rate (ESR). Bioinformatic analysis linked the differentially expressed proteins primarily to inflammatory mechanisms. These findings offer crucial molecular insight into J-Gout pathogenesis and serve as promising diagnostic or therapeutic biomarkers. Following the ionic analysis, this proteomic exploration demonstrates how molecular communication via exosomes encodes disease-specific inflammatory signatures.

Wang et al. analyzed the time-dependent biological variation (BV) of 16 biomarkers related to thyroid function, iron metabolism, and bone metabolism in 24 stable Type-2 Diabetes Mellitus (T2DM) patients. They also used variation values derived from healthy subjects, showing that some markers could be precisely monitored in T2DM patients by applying these reference change values. Conversely, for certain biomarkers, personalized monitoring was emphasized over using variation derived from healthy groups. This study illustrates the transition from individual molecular measures to dynamic systems of integrated biomarkers, reinforcing the need for personalized interpretation in metabolic diseases.

Wang and Zhu applied a two-sample Mendelian randomization (MR) analysis—an influential method for causal inference developed in the early 2000s (2)—using large-scale GWAS summary data comprising 1,195 rosacea cases and 211,139 controls to investigate the causal relationships between 179 plasma lipid species and rosacea. Two sterol esters (SE), two phosphatidylethanolamines (PE), and one sphingomyelin (SM) were identified as statistically significant protective factors against rosacea risk. This research enhances the understanding of rosacea pathogenesis by suggesting that these lipids are crucial for maintaining cell membrane function and regulating immune responses. It represents novel molecular targets for assessing and potentially treating this dermatological condition. Their work connects biochemical variability with genetic causality, demonstrating how lipid species can bridge metabolism, immunity, and dermatological pathology.

Ke et al. advanced the field of diagnostic marker discovery by applying bulk RNA analysis integrated with a comprehensive bioinformatics workflow - including differentially expressed gene (DEG) analysis, weighted gene co-expression network analysis (WGCNA), and machine learning - to identify potential diagnostic genes in patients with Diabetes Mellitus (DM). They further highlighted the biological significance by noting its strong correlation with variations in immune cell types, suggesting a pivotal role in DM’s immunoregulatory mechanisms. This work leverages transcriptomic networks and machine learning to map the immune-metabolic landscape of endocrine disease.

Buzdin et al. introduced the EndoGene database, which is a repository documenting genetic variants identified via NGS and WES in 5,926 Russian patients with endocrine disorders. This work is valuable and meaningful from both an ethnic and population genetics perspective. This database is vital from a population-specific perspective due to the genetic heterogeneity of the Russian Federation. The study reported 2,073 unique genetic variants, with a striking 57% being previously undescribed at the time of genetic interpretation. EndoGene contributes essential population statistics and genetic background information, aiding clinicians in interpreting rare or population-specific mutations and ultimately enhancing the diagnostic accuracy and informative power of clinical NGS panels for endocrine pathologies. In the broader context, this database serves as an anchor point for genomic diversity, ensuring that future biomarker interpretation reflects population-specific genetic architecture.

Zhang et al. extended the concept of the Pan-Immune-Inflammation Value (PIV) - a composite biomarker integrating neutrophils, platelets, monocytes, and lymphocytes, originally proposed around 2020 as a prognostic indicator in cancer patients (3) - to broader applications encompassing general disease and mortality outcomes. They evaluated PIV as a predictor of mortality in the general population from a nationwide cohort study (NHANES, 48,662 samples). They found PIV levels were significantly and independently associated with an increased risk of all-cause mortality, as well as cause-specific deaths (cardiovascular, cancer, and diabetes-related). Moreover, a significant nonlinear dose-response relationship was observed between PIV and all-cause, cardiovascular disease, and cancer mortality. This research supports PIV’s utility for public health risk stratification. Following the molecular and genomic studies, this large-scale investigation illustrates how integrated immune indices can extend biomarker discovery to population-level prediction.

Zhang et al. retrospectively analyzed 420 Chinese pregnant women with preeclampsia (PE) who had concomitant gestational hypothyroidism (GHT) to investigate the complex association between PE/GHT and neonatal birth weight (BW). Neonates born to mothers suffering from both PE and GHT exhibited significantly lower birth weight compared to those born to women with PE alone. Crucially, maternal Alanine Aminotransferase (ALT) levels, which were significantly elevated in the PE/GHT group, were identified as a potential partial mediator in this relationship. This highlights the necessity for clinicians to closely monitor maternal thyroid and liver function in PE patients to improve neonatal outcomes. Positioned toward the conclusion, their work exemplifies system-level biomarker integration, linking endocrine, hepatic, and obstetric parameters to clinical outcomes.

Cao et al. investigated the characteristics of the gut microbiome in 30 patients with Asymptomatic Hyperuricemia (AH) compared to 30 healthy controls using 16S rRNA sequencing. The AH group exhibited decreased overall gut microbial richness and ecological diversity. These microbial changes offer new insights and suggest that specific species may serve as potential biomarkers for early diagnosis and monitoring of AH. As the final piece, this study completes the integration spectrum by linking internal endocrine metabolism to external ecological networks, emphasizing that precision endocrinology now extends beyond the human genome into the microbiome.

Together, these nine contributions delineate a rapidly expanding frontier in integrated diagnostics, spanning the full continuum of biological organization—from ionic ratios and proteomic signatures to genomic databases and microbiome-derived ecological biomarkers. Collectively, they illustrate how endocrine science is evolving from isolated molecular characterization toward a fully systems-based discipline, in which the integration of multi-omics, clinical, and environmental data enhances both mechanistic insight and translational precision.

This convergence reflects the maturation of data-informed endocrinology, where diagnostic and prognostic innovation emerges from the synthesis of diverse data modalities rather than from any single layer of observation. By harmonizing biochemical, genetic, immunologic, and ecological perspectives, these studies redefine biomarker discovery as a process of multi-scale inference and integration - one that connects molecular precision with population-level relevance and real-world applicability. In this new framework, integration is not merely a methodological approach but a scientific imperative - transforming endocrinology into a discipline that systematically bridges molecules to medicine, and data to diagnosis.
